# Clinical Features and Response to Treatment in Elderly Subjects Affected by Hidradenitis Suppurativa: A Cohort Study

**DOI:** 10.3390/jcm12247754

**Published:** 2023-12-18

**Authors:** Flaminia Antonelli, Elena Ippoliti, Elia Rosi, Chiara Moltrasio, Dalma Malvaso, Elisabetta Botti, Damiano Abeni, Valentina Dini, Maria Vittoria Cannizzaro, Manfredo Bruni, Lucia Di Nardo, Maria Concetta Fargnoli, Marco Romanelli, Luca Fania, Luca Bianchi, Angelo Valerio Marzano, Francesca Prignano, Ketty Peris, Andrea Chiricozzi

**Affiliations:** 1 Dermatologia, Dipartimento Universitario di Medicina e Chirurgia Traslazionale, Università Cattolica del Sacro Cuore, 00168 Rome, Italy; flamiantonelli@gmail.com (F.A.); elena.ippoliti01@gmail.com (E.I.); luciadinardo@hotmail.it (L.D.N.); ketty.peris@unicatt.it (K.P.); 2 U.O.C. Dermatologia, Dipartimento di Scienze Mediche e Chirurgiche, Fondazione Policlinico Universitario A. Gemelli IRCCS, 00168 Rome, Italy; malvasodalma@gmail.com (D.M.); mariavittoria.cannizzaro@gmail.com (M.V.C.); 3 Section of Dermatology, Department of Health Sciences, University of Florence, 50121 Firenze, Italy; elia.rosi@unifi.it (E.R.); francesca.prignano@unifi.it (F.P.); 4 Dermatology Unit, Fondazione IRCCS Ca’ Granda Ospedale Maggiore Policlinico, 20122 Milan, Italy; chiara.moltrasio@policlinico.mi.it (C.M.); angelo.marzano@unimi.it (A.V.M.); 5 Department of Pathophysiology and Transplantation, Università degli Studi di Milano, 20122 Milan, Italy; 6 Department of Systems Medicine, University of Rome “Tor Vergata”, 00133 Rome, Italy; elisabetta.botti@uniroma2.it (E.B.); luca.bianchi@uniroma2.it (L.B.); 7 Dermatology Unit, Fondazione Policlinico “Tor Vergata”, 00133 Rome, Italy; 8 Istituto Dermopatico dell’Immacolata, IDI-IRCCS, Dermatological Research Hospital, 00167 Rome, Italy; d.abeni@idi.it (D.A.); l.fania@idi.it (L.F.); 9 Unit of Dermatology, University of Pisa, 56126 Pisa, Italy; valentina.dini@unipi.it (V.D.); marco.romanelli@unipi.it (M.R.); 10 Department of Biotechnological and Applied Clinical Sciences, University of L’Aquila, 67100 L’Aquila, Italy; manfredo.bruni@gmail.com (M.B.); mariaconcetta.fargnoli@univaq.it (M.C.F.); 11 Dermatology Unit, Ospedale San Salvatore, 67100 L’Aquila, Italy

**Keywords:** hidradenitis suppurativa, elderly, Hurley stage, phenotype, fistulas

## Abstract

Hidradenitis suppurativa (HS) is a chronic-relapsing inflammatory skin disease. It usually appears in the second and third decades, but a smaller proportion of patients develop late-onset HS. Geriatric HS, defined as the persistence or the development of HS after the age of 65 years, has been poorly explored. This study aimed to investigate the clinical features, treatment management and response to therapies of HS elderly subjects (≥65 years old). We designed a multicentric observational study, gathering data from seven Italian university hospitals. Demographic and clinical data of HS patients aged over 65 years were collected at baseline, week 12 and week 24. Overall, 57 elderly subjects suffering from HS were enrolled. At baseline, disease severity was predominantly moderate-to-severe, with 45.6% of patients classified as Hurley III. The gluteal phenotype was the most frequently observed; it also appeared to affect patients’ quality of life more than other phenotypes. Gluteal involvement was detected in about half (49.1%) of cases and associated with severe stages of the disease. In terms of therapeutic response, Hurley III patients showed the persistency of higher values of mean IHS4, DLQI, itch- and pain-NRS scores compared to Hurley I/II. In conclusion, disease severity in this subpopulation appears high and treatment is often challenging.

## 1. Introduction

Hidradenitis suppurativa (HS) is a chronic-relapsing inflammatory skin disease affecting less than 1% of the general population [1]. It is clinically characterized by the development of recurrent, painful, deep-seated, inflammatory nodules, abscesses and fistulas, mostly localized in typical body areas, such as skin folds, gluteal and genital sites. HS has a marked female predominance, with a female-to-male ratio exceeding 2:1 in the U.S. population [2]. Interestingly, prevalence tends to decline in postmenopausal women [3], probably related to the decrease of hormonal triggers. The onset of HS usually occurs in young adulthood, with clinical signs that usually appear after puberty, in the second and third decades [4]. Specifically, most patients (76%) present with early-onset HS, peaking at 17 years of age, while a smaller proportion of individuals develop late-onset HS, peaking at around 40 years of age [5]. Geriatric HS, defined as the persistence of active disease beyond the age of 65 years or the development of HS occurring after that age, has been poorly investigated, with only a few case reports and cohort studies published so far [6,7,8,9,10,11]. The aim of this multicentric, retrospective study was to investigate the clinical features of HS in elderly subjects and to identify peculiar aspects related to treatment management and response to therapies.

## 2. Materials and Methods

Patients aged ≥65 years who suffered from HS were included in this multicentric observational study conducted in seven Italian University dermatology departments. After protocol approval by the Local Ethics Committee—Comitato Etico Territoriale (CET) Lazio Area 3, Prot. ID: 5865, data were collected both retrospectively and prospectively, through the period ranging from January 2022 to April 2023. Prior to study enrollment, patients provided written informed consent.

From patient charts, we collected demographic data (age, gender, weight, height, BMI, smoking status) and clinical data at baseline, including details on family history of HS, disease severity and clinical phenotype (Canoui-Poitrine phenotype, Hurley stage, IHS4 (International Hidradenitis Suppurativa Severity Score System), DLQI (Dermatology Life Quality Index) score, pain-NRS score, itch-NRS score, lesion type, total number of lesions and their localization, age at disease onset, disease duration meant as years passed from diagnosis to study enrollment, previous treatments for HS). The Canoui-Poitrine classification was used to categorize the clinical presentation of HS into three different phenotypes according to the main areas affected and prevalent skin lesion type: (1) axillary–mammary involvement, which mainly affects females; (2) the follicular subtype associated with acne and usually affecting males and the gluteal variant [12]. We also collected data on comorbidities, concomitant medications and previous treatments for HS. At the follow up visits, scheduled at week 12 and week 24, clinical data were registered using the same assessment scores used at baseline. Data recorded by each center were subsequently merged to allow data analysis.

To describe the therapeutic management, patients were divided into 5 subgroups based on the main therapy: (1) topical treatment (antibiotics/steroids), (2) systemic antibiotics, (3) biological treatment (anti-TNF), (4) systemic corticosteroids and (5) dapsone. Systemic antibiotics were further subclassified in 3 groups, according to international guidelines: monotherapy with antibiotics (such as tetracyclines and clindamycin), combination of antibiotic treatments (clindamycin and rifampicin), monotherapy with antibiotics not in treatment guidelines but proposed for selective cases by the physician (e.g., azithromycin, ciprofloxacin).

To assess disease activity, International Hidradenitis Suppurativa Scoring System (HIS4 score) was used. IHS4 scoring derives from the sum of the skin lesions, considering a value of 1 for each inflammatory nodule, 2 for each abscess and 4 for each fistula [13]. According to HIS4 thresholds identifying mild (IHS4 ≤ 3), moderate (IHS4 4–10) and severe (IHS4 ≥ 11) forms of disease [14], in our analysis, patients showing HIS4 < 11 were grouped into a mild-to-moderate disease cluster, while those showing IHS4 ≥ 11 were grouped in the severe disease cluster. The Dermatology Life Quality Index (DLQI) [15] was used to evaluate each patient’s health-related quality of life at each visit. This questionnaire is composed by 10 items evaluating different aspects of life quality impairment in the last 7 days before performing the test. Each question scores 0 to 3, with a maximum total score of 30, which indicates the greatest impairment of quality of life [16].

### Statistical Analyses

Descriptive statistic was carried out by presenting categorical data as frequency distribution and continuous data as median (interquartile range) or mean (Standard Deviation, SD), appropriately. Disease and patients’ characteristics were stratified according to Hurley staging—considering each stage independently and dividing patients with Hurley I/II from those with Hurley III—and IHS4, defining severe HS by IHS4 ≥ 11 and mild-to-moderate by IHS4 < 11. In particular, we dissected the study population according to disease onset (early onset ≤ 50 versus late onset > 50 years), analyzing any potential difference in terms of mean BMI, prescription of systemic immunomodulatory therapies or the development of type II diabetes.

Multivariable regression models were designed using the backward elimination procedure to identify factors that could influence the treatment choice. Coefficient values for the regression equation were estimated (with 95% confidence intervals) for the independent variables (type of treatment choice) as predictors of the dependent variables (itch- and pain-NRS, IHS4 and DLQI). All statistical tests were two-sided, and a *p*-value of ≤0.05 was considered statistically significant. Statistical analysis was performed using Stata/BE 18.0 (StataCorp LLC, 4905 Lakeway Drive, College Station, TX, USA).

## 3. Results

Overall, 57 patients suffering from HS and aged over 65 years were included. Most of the patients were females (35/57, 61.4%) and active smokers (34/57, 59.7%; Table 1).

At baseline, the mean (±SD) patient age was 69.8 (±4.9) years, with a mean age at HS onset of 49.5 (±16.7) years and a mean disease duration of 20.3 (±15.9). Six of 57 patients (10.5%) reported a disease onset beyond 65 years old. Family history of HS was uncommon, being reported in 10.5% (6/57) of cases. The patient cohort showed a mean (±SD) BMI of 28.2 (4.9), with 9/44 (20.4%) of cases affected by obesity (BMI ≥ 30). Dissecting the study population according to disease onset (early onset [age ≤ 50 years] versus late onset [age > 50 years]), we found no significant differences between these two subcohorts in terms of BMI (early onset: 29.3 ± 5.9 versus late onset: 27.6 ± 4.2, *p* = 0.286), systemic immunomodulant medications (early onset: 8.3%(2/24) versus late onset: 13.6% (3/22), *p* = 0.564) or the development of type II diabetes (early onset: 12.0%(3/25) versus late onset: 28.1% (9/32), *p* = 0.138). Comorbidities were present in 53/57 (92.9%) patients, with cardiovascular and metabolic disorders reported in 32/53 (60.4%), immuno-mediated disease in 9/53 (16.9%) and pulmonary conditions in 5/53 (9.4%). Out of 57, 12 patients (21.1%) were affected by type II diabetes at the time of study enrollment. Comorbid conditions commonly occurred prior to HS onset, such as diabetes (n. 2 cases) and gain of weight (n. 3 cases). Specifically, one patient had diabetes onset and one patient had diabetes worsening within 6 months before HS onset. One patient had weight gain of over 10 Kg and two patients had unspecified weight gain within 6 months prior to HS onset, with one patient with weight gain who experienced HS worsening after the increase of body weight. Also, the concomitant use of potentially aggravating medications or the occurrence of inborn error of immunity was examined: one patient started rituximab and one patient started dasatinib therapy prior to HS onset. Another patient underwent methotrexate and cyclosporine therapy and one patient had systemic corticosteroids prior to disease onset. In these cases, medications were started less than 6 months prior to HS onset. The most frequently observed clinical phenotype, according to the Canoui-Poitrine classification, was the gluteal phenotype as assessed in 23/57 (40.3%) patients, including 13 males and 10 females, followed by the axillary–mammary (20/57, 35.1%) and follicular (14/57, 24.6%) phenotypes. Pelvic MRI was not routinely prescribed for all patients with gluteal lesions, but it was prescribed case by case according to extension and type of involvement of the perianal area. Some patients were prescribed MRI, but they did not receive it, while one patient was prescribed MRI that revealed the presence of a complex fistula. Overall, in our population, perianal fistulas were found in three patients. Most patients exhibited inflammatory nodules (48/57, 84.2%), abscesses (40/57, 70.2%) and fistulae (36/57, 63.2%), but comedones and hypertrophic scars were also detected (25/57, 43.9%, for both types of lesions).

At baseline, disease severity was mainly assessed as moderate-to-severe, with 26 patients (45.6%) classified as Hurley III, 21 (36.8%) as Hurley II and 10 (17.6%) with mild manifestations (Hurley I). Mean baseline IHS4 and DLQI scores were 14.6 (±13.7) and 11.3 (±8.9), respectively. Nevertheless, symptom intensity was relatively mild, with a mean pain-NRS score of 5.2 (±3.5) and mean itch-NRS score of 2.6 (±2.6). According to the IHS4 scoring system, 28 patients (49.1%) had severe HS (defined by IHS4 ≥ 11), exhibiting higher mean BMI compared with patients showing IHS4 < 11 (29.7 (5.6) versus 26.6 (3.4), *p* = 0.039) and more frequent gluteal phenotype (16/28, 69.6%, versus 7/29, 30.4%, *p* = 0.032). Consistently, gluteal phenotype was also more often reported in Hurley III patients (17/26, 65.4%) compared with the Hurley II (9/21, 42.9%) and Hurley I disease stages (2/10, 20%, *p* = 0.039; Table 1).

In addition, the HS phenotype appears to significantly affect patients’ quality of life, as gluteal phenotype was associated with a significantly higher DLQI score (16.2 ± 9.6) than axillary–mammary (8.8 ± 7.3) or follicular (6.2 ± 5.5) phenotypes (analysis-of-variance *p* = 0.005).

On the contrary, the number (Pearson’s correlation *p* = 0.644) and type of comorbidities did not affect disease severity based on IHS4 scoring.

### Therapeutic Management of Elderly HS Patients

A total of 41 patients (71.9%) received any systemic therapy, while 16 (28.1%) were treated with topical treatment only. Among those undergoing a systemic therapy, most (75.6%, 31/41) received antibiotics (mainly rifampicin plus clindamycin, doxycycline, azithromycin, amoxicillin, tetracyclines, ciprofloxacin), while four (9.8%) were treated with adalimumab. Patients with Hurley III staging were more frequently treated with systemic antibiotics (69.2%, 18/26) compared with Hurley I (20%, 2/10) and II (52.4%, 11/15; *p* = 0.012). Overall, 12 out of 57 patients showed concomitant comorbidities representing a contraindication or discouraging the use of biological therapy, mainly cardiovascular diseases (9/12, 75%), latent tuberculosis (1 case), non-Hodgkin lymphoma (1), myeloid leukemia (1) and squamous cell carcinoma (1).

Investigating disease and patient characteristics that could influence treatment choice, the DLQI and itch-NRS score were identified as critical factors. Systemic antibiotics or corticosteroids were most commonly prescribed to patients with higher DLQI scores (Prob > F = 0.0003, R-squared = 0.3358), while systemic corticosteroids were most commonly prescribed than topical treatment to patients with higher itch-NRS (Prob > F = 0.0082, R-squared = 0.2632). A short-term course of oral prednisolone at the dosage of about 0.2–0.5 mg/Kg daily with a progressive decrease of the dosage within one month was commonly prescribed. Disease severity (Prob > F = 0.1603, R-squared = 0.1167), pain-NRS (Prob > F = 0.0614, R-squared = 0.1560), disease phenotypes (Pearsons chi2 = 0.062), anatomical disease locations (Folds: Pearsons chi2 = 0.763; perineal area: Pearsons chi2 = 0.371; gluteal: Pearsons chi2 = 0.261; other sites: Pearsons chi2 = 0.544) and patient BMI (Prob > F = 0.228, R-squared = 0.1345) did not appear to influence the therapeutic choice. We assessed treatment effectiveness and quality of life for those patients observed through 24 weeks. A slight improvement of disease severity was detected in terms of reduction of IHS4, DLQI, pruritus- and pain-NRS scores from baseline through the observation period (Table 2). To further investigate potential factors affecting the therapeutic response to HS-dedicated treatments, we considered the concomitant or prior use of immunomodulant medications. At the time of study enrollment, prior use of systemic corticosteroids and/or immunomodulant agents (cyclosporin, methotrexate, dapsone, azathioprine, rituximab, salazopirine, dasatinib) was referred by 5 out of 57 patients (10.9%). At the baseline, 12 out of 57 patients (21.1%) started treatment with systemic corticosteroids and/or immunomodulant agents. In addition, four patients had systemic steroids and/or immunosuppressive medications (methotrexate, cyclosporine, rituximab, dasatinib) within 6 months prior to HS onset. Comparing patients with or without concomitant or prior use of systemic steroids/immunomodulant therapies, we did not detect any significant difference at week 12 and week 24, in terms of therapeutic response (reduction of IHS4, DLQI pain-NRS, itch-NRS, and HISCR50 scores), except for a greater amelioration of IHS4 (*p* = 0.009) and pain-NRS (*p* = 0.014) after 24 week of treatment among those patients who had prior use of systemic corticosteroids/immunomodulant agents (Supplementary Table S1).

Overall, mean values (±SD) of IHS4 and DLQI of 9.3 (±2.2) and 9.1 (±1.5), respectively, revealed residual disease manifestations assessed as moderate after 24 weeks of treatment (Table 2). However, when the study population was stratified in two groups according to disease staging (Hurley I/II versus Hurley III), patients with Hurley III disease showed the persistency of significantly higher values of mean IHS4, DLQI, itch- and pain-NRS scores than patients with Hurley I/II disease, despite a 24-week treatment (Table 3). At week 24, nine out of 29 (31.0%) observed patients still presented a severe form of HS (IHS4 ≥ 11).

## 4. Discussion

A limited body of evidence defining clinical and demographic features of elderly subjects affected by HS is currently available. This multicentric study defined a peculiar clinical profile of elderly HS patients showing a high mean age of onset (52.4 years), in agreement with a bimodal distribution of age of HS onset [5,17,18]. The elevated mean age of onset in the elderly was also detected by a recent study describing a higher age of onset in elderly patients (≥65 years old) affected by HS compared with a younger cohort [6]. We identified elderly patients as those subjects of ≥65 years old, though Van der Weijden et al. proposed to identifiy “HS tarda” in patients aged over 60 years, differentiating between persistent HS tarda, appearing before 60 years of age, and late-onset HS tarda, developing after this age. Based on this age cutoff, the prevalence of HS was investigated through a specific questionnaire conduced in the Northern Netherlands and involving 56,084 subjects [7]. About 1000 cases of active HS were found, including 209 patients aged over 60 years. The resulting prevalence of HS in a general population older than 60 years old was 1%, consisting of 0.8% of persistent HS and 0.2% of late-onset HS [7]. Moreover, this study confirmed a higher age of onset and a higher proportion of males among elderly subjects compared to the adult subcohort, possibly due to the influence of menopause on the HS course. In addition, the elderly patient subcohort was more likely affected by comorbidities than the adult counterpart, including history of acne, diabetes, psoriasis, history of PCOS and others [7].

Few cases of positive family history for HS were observed in our population, in contrast to the literature data describing 30–40% of patients having at least one affected family member [19,20]. This could pave the way for the hypothesis of a greater importance of environmental factors compared to genetic factors in geriatric HS. The gluteal phenotype was the predominant phenotype, conversely to the phenotype pattern distribution described in the general HS population, who are mostly affected by the axillary–mammary phenotype (48% of HS patients), while the follicular and gluteal phenotypes were reported in 26% of cases for both [21]. Moreover, the national Italian registry IRHIS showed that the gluteal area was affected in 29.7% of patients, whereas the groin was involved in 70.7% and the axillae in 61.8% of participants [22]. On the contrary, in our patient cohort, gluteal involvement was detected in about half of cases (28/57, 49.1%) and appeared to be associated with severe stages of the disease (65.4% of gluteal cases were classified as Hurley 3 stage). This finding is in contrast with a previous study conducted by Canoui-Poitrine et al. describing a milder disease in the gluteal phenotype compared with the axillary–mammary phenotype [12] Interestingly, in this study, the gluteal class was also associated with a longer duration of disease, which could suggest a link to the high prevalence in advanced age observed in our population [12].

Our study revealed a high degree of severity and a low response to treatment in elderly HS patients. Severe cases, classified as Hurley III or IHS4 ≥ 11, constituted 45.6% or 49.1% of all elderly HS cases, respectively. The elevated rate of severe cases is in line with previous studies, and it was associated with a high DLQI score [18]. The higher degree of disease severity in elderly patients (odds ratio [OR], 8.7; 95% CI, 2.5–29.8) was detected in a previous study when compared with younger adults (OR, 3.6; 95% CI, 2.6–4.8) [6]. Notably, disease severity was not markedly dampened by the prescribed therapies within 24 weeks of observation. Indeed, a suboptimal control of the disease was detected as a reduction of disease severity scores was obtained with a still-high residual disease activity (mean IHS4 of 9.3 and mean DLQI of 9.1 after 24 weeks of treatment). In particular, severe patients classified at the baseline as Hurley III appeared resistant to treatment, obtaining an inadequate response to treatment. They maintained high values of severity scores after 12 and 24 weeks of therapy. The elevated severity and the persistency of a high degree of disease activity in elderly HS patients could represent the result of multiple concomitant aspects. Firstly, comorbidities (such as heart failure for anti-TNF antibodies and renal or hepatic failure for antibiotics) and frailty might affect treatment choice, constituting contraindications to or discouraging the use of immunomodulant drugs. Another class of agents whose use could be limited in elderly subjects is systemic retinoids; these represent a valid treatment option but, due to comorbidities requiring a strict control of blood cholesterol levels and to multiple comedications, frequently owing to liver metabolism, their use might be discouraged. Moreover, elderly subjects often exhibit dryness of the skin and mucous membranes, with increased risk of epistaxis, which could worsen with retinoid therapy. A careful selection of some concomitant medications, particularly those potentially aggravating or revealing an inborn error of immunity that might be associated with HS occurrence, should be considered [23,24]. This could lead to the selection of the safest choice but not the most effective one, resulting in an undertreatment or suboptimal care. Older people often have barriers to health care hub access and therefore might underestimate initial clinical manifestations because of a self-misperception of the disease. The severe clinical manifestations of HS in elderly patients might be related to the fact that older adults may have a longer duration of disease, leading to the development of scar tissue and fistulas, which further provide a substrate for the perpetuation of inflammation and represent a negative factor contributing to a poor response to treatment [25,26]. The presence of associated anal fistulas may influence severity and resistance to treatment of patients with gluteal involvement, but our data are not sufficient to have a demonstration of this hypothesis. Lastly, a diagnostic delay and delayed referral may occur [27,28]. Despite the low prevalence of HS in advanced age, the characterization of elderly patients (≥65) has no negligible clinical relevance. Disease severity in this subpopulation appears high, and treatment is often challenging. Notably, the gluteal localization of cutaneous lesions was associated with a greater impact on quality of life and elevated disease activity. This study reported data from a heterogeneous population in terms of prescribed therapies accounting for different therapeutic approaches adopted by each physician, with a number of patients that limited statistical analyses, despite the fact that our study population is the relatively largest real-world elderly population to be investigated. Additionally, this study provides insights about a patient population, the elderly one, that is excluded from or shows limited access to clinical trials; therefore, further real-world experiences are needed for a deeper understanding of the clinical features and treatment response of elderly HS patients. Moreover, the late-onset subgroup might be the right setting to explore additional trigger factors for HS onset, such as diabetes, meaningful weight gain and use of immunosuppressive/immunomodulant medications. Prospective studies of large cohorts are needed to analyze the real contribution of these factors in the development of HS, especially during the 6 months preceding HS onset. Moreover, prospective studies exploring clinical features and treatment response in patients with perianal fistulas compared to patients without perianal fistulas could also provide clinically meaningful evidence.

## 5. Conclusions

Our study suggests that HS does not affect exclusively young subjects, but it may last or appear in elderhood, representing an even more challenging condition because the therapeutic approach might be limited by the higher number of comorbidities, comedications and the frailty of this specific subpopulation. Thereby, further studies are needed to better define the clinical profile and a tailored treatment paradigm for elderly subjects affected by HS.

## Figures and Tables

**Table 1:** Clinical characteristics of the overall study population and according to Hurley staging.

Characteristics of Patients		Overall Study Population (N = 57)	Hurley I (n = 10)	Hurley II (n = 21)	Hurley III (n = 26)	*p* Value
Gender	Male n (%)	22 (38.6)	2 (20.0)	7 (33.3)	13 (50.0)	0.209
	Female n (%)	35 (61.4)	8 (80.0)	14 (66.7)	13 (50.0)	
Age at disease onset [mean(±SD)] (years)		49.5 (16.7)	49.1 (15.3)	48.1 (21.2)	50.7 (13.2)	0.870 *
Age at study enrollment [mean(±SD)] (years)		69.8 (4.9)	68.9 (3.3)	70.7 (6.5)	69.5 (4.0)	0.584 *
BMI [mean(±SD)]		28.2 (4.9)	27.1 (4.1)	27.6 (3.8)	29.2 (5.9)	0.479 *
Family history of HS	Yes n (%)	6 (10.5)	0 (0.0)	3 (50.0)	3 (50.0)	0.468
	No n (%)	51 (89.5)	10 (19.6)	18 (35.3)	23 (45.1)	
Smoking	Yes n (%)	34 (59.7)	7 (70.0)	11 (52.4)	16 (61.5)	0.645
	No n (%)	14 (24.6)	1 (10.0)	6 (28.6)	7 (26.9)	
	Ex-smoker n (%)	9 (15.8)	2 (20.0)	4 (19.0)	3 (11.6)	
Comorbidities	Yes n (%)	53 (92.9)	8 (80.0)	20 (95.2)	25 (96.2)	0.207
	Cardiovascular and metabolic (hypertension, cardiopathy, diabetes type I, dyslipidemias)	32 (60.0)	6 (18.7)	11 (34.4)	15 (46.9)	0.552
	Chronic Pulmonary (BPCO)	5 (9.4)	0	3 (37.5)	5 (62.5)	0.212
	Psychiatric (anxiety/depression)	4 (10.0)	1 (25.0)	1 (25.0)	2 (50.0)	0.921
	Immuno-mediated (arthritis, thyroiditis, pemphigus, psoriasis, lichen, chronic pancreatitis)	9 (16.9)	2 (20.0)	4 (19.0)	3 (11.5)	0.721
	Current Infection (latent tuberculosis)	1 (2.5)	0	0	1 (100.0)	0.343
	History of/or current cancer	10 (17.5)	3 (30.0)	1 (4.8)	6 (23.1)	0.136
Dominant/Prevalent treatment	Topical (antibiotic/steroids)	16 (28.1)	8 (80.0)	6 (28.6)	2 (7.7)	0.012
	Systemic antibiotic	31 (54.4)	2 (20.0)	11 (52.4)	18 (69.2)	
	Biological (adalimumab)	4 (7.0)	0 (0.0)	1 (4.8)	3 (11.5)	
	Systemic corticosteroids	2 (3.5)	0 (0.0)	1 (4.8)	1 (3.9)	
	Dapsone	4 (7.0)	0 (0.0)	2 (9.4)	2 (7.7)	
Disease features						
Disease duration [mean(±SD)] (years)		20.3 (15.9)	19.8 (17.1)	22.6 (19.0)	18.8 (13.0)	0.726 *
Phenotype	Axillary/mammary;	20 (35.1)	5 (50.0)	7 (33.3)	8 (30.8)	0.180
	Follicular	14 (24.6)	4 (40.0)	6 (28.6)	4 (15.4)	
	Gluteal	23 (40.3)	1 (10.0)	8 (38.1)	14 (53.9)	
Anatomical site involvement	Folds	46	7 (70.0)	18 (85.7)	21 (80.8)	0.584
	Perineal area	28	4 (40.0)	8 (38.1)	16 (61.5)	0.090
	Gluteal	28	2 (20.0)	9 (42.9)	17 (65.4)	0.039
	Other	9	0	0	3 (15.8)	0.120
IHS4 [mean(±SD)]		14.6 (13.7)	4.1 (2.7)	7.9 (5.5)	24.1 (14.8)	<0.0001 *
DLQI [mean(±SD)]		11.3 (8.9)	2.2 (2.7)	7.0 (5.4)	18.0 (7.5)	<0.0001 *
Pain-NRS [mean(±SD)]		5.2 (3.5)	2.2 (3.0)	3.8 (3.2)	7.6 (2.2)	<0.0001 *
Itch-NRS [mean(±SD)]		2.6 (2.6)	1.1 (1.6)	1.4 (1.6)	4.0 (2.8)	0.0007 *

* Analysis of variance (ANOVA).

**Table 2:** Disease severity assessment through an observation period of 24 weeks.

Overall Population	Baseline	Week 12 (N = 36)	*p* Value *	Week 24 (N = 29)	*p* Value §
IHS4 [mean(±SD)]	14.3 (2.1)	10.6 (1.9)	<0.0001	9.3 (2.2)	<0.0001
DLQI [mean(±SD)]	12.7 (1.6)	10.4 (1.6)	0.0051	9.1 (1.5)	0.0004
Itch-NRS [mean(±SD)]	5.7 (3.5)	4.6 (3.4)	0.035	3.5 (3.1)	0.0002
Pain-NRS [mean(±SD)]	2.5 (2.4)	2.2 (2.2)	0.039	1.3 (1.9)	0.0001
HISCR50		13/36 (36.1%)		12/29 (31.0%)	

* *p* value for the comparison between baseline and w.12 (paired *t* test). § *p* value for the comparison between baseline and w.24 (paired *t* test).

**Table 3:** Disease severity assessed at the baseline and follow-up visits, according to disease staging (severe versus mild-to-moderate).

	Hurley I/II			Hurley III		
	Baseline (n = 31)	w.12 (n = 17)	w.24 (n = 11)	Baseline (n = 26)	w.12 (n = 19)	w.24 (n = 18)
IHS4 * [mean(±SD)]	6.7 (5.0)	3.9 (2.4)	3.2 (2.8)	24.1 (14.8)	16.6 (15.8)	13.1 (13.7)
DLQI ^ç^ [mean(±SD)]	5.3 (5.1)	4.7 (5.0)	4.6 (3.3)	18.0 (7.5)	15.1 (9.4)	11.9 (8.5)
Pain-NRS ° [mean(±SD)]	3.2 (3.2)	2.7 (2.6)	2.0 (2.0)	7.6 (2.2)	6.3 (3.2)	4.4 (3.3)
Itch-NRS ^§^ [mean(±SD)]	1.3 (1.6)	1.4 (1.4)	0.7 (1.0)	4.0 (2.8)	2.9 (2.6)	1.6 (2.2)

* Comparison of IHS4 between Hurley I/II and Hurley III groups. Baseline IHS4: *p* < 0.0001; w12: *p* = 0.0007; w24: *p* = 0.0264. ^ç^ Comparison of DLQI between Hurley I/II and Hurley III groups. Baseline DLQI: *p* < 0.0001; w.12: *p* = 0.0004; w.24: *p* = 0.0118. ° Comparison of pain-NRS between Hurley I/II and Hurley III groups. Baseline pain-NRS: *p* < 0.0001; w.12: *p* = 0.0008; w24: *p* = 0.0369. ^§^ Comparison of itch-NRS between Hurley I/II and Hurley III groups. Baseline itch-NRS: *p* = 0.0001; w.12: *p* = 0.0418; w.24: *p* = 0.2079.

## Data Availability

Enquiries related to the data generated or analyzed during this study can be directed to the corresponding author.

## Supplementary Materials

The following supporting information can be downloaded at: https://www.mdpi.com/article/10.3390/jcm12247754/s1, Table S1: Therapeutic response by dissecting the study population into two patient subcohorts according to the concomitant or prior use of systemic steroids/immunomodulant therapies.
