# Supplementary material for: Clinical Features and Response to Treatment in Elderly Subjects Affected by Hidradenitis Suppurativa: A Cohort Study
Source: J Clin Med. 2023 Dec 18;12(24):7754. doi: 10.3390/jcm12247754 (PMC10744325; doi:10.3390/jcm12247754)
Supplement: Supplementary file 1 [file jcm-12-07754-s001.zip › jcm-2737091-supplementary.pdf]

Supplementary Table S1. Therapeutic response by dissecting the study population into two patient subcohorts according to the concomitant or prior use of systemic steroids/immunomodulant therapies.

**Week 12**	Patients referred prior use of immunomodulant (n=5)	NO prior use of immunomodulant (n=41)	P value
IHS4 difference°[mean(±SD)]	-4.3(3.3)	-3.8(3.5)	0.823
DLQI difference°[mean(±SD)]	-4.5(7.3)	-2.1(4.2)	0.346
Pain- NRS difference°[mean(±SD)]	-1.3(1.9)	-1.1(2.2)	0.919
Itch- NRS difference°[mean(±SD)]	-0.25(1.7)	-0.3(0.8)	0.862
HISCR50 n(%)	1(25.0)	12(40.0)	0.562
	Current use of immunomodulant (n=12)	NO current use of immunomodulant (n=45)	P value
IHS4 difference°[mean(±SD)]	-3.9(4.1)	-3.6(3.3)	0.891
DLQI difference°[mean(±SD)]	-1.1(5.9)	-2.6(4.2)	0.463
Pain- NRS difference°[mean(±SD)]	-0.57(1.1)	-1.2(2.2)	0.475
Itch- NRS difference°[mean(±SD)]	-0.43(0.86)	-0.3(0.9)	0.678
HISCR50 n(%)	2(28.6)	11(37.9)	0.644
**Week 24**	Patients referred prior use of immunomodulant (n=5)	NO prior use of immunomodulant (n=41)	P value
IHS4 difference°[mean(±SD)]	-15.7(5.7)	-6.9(4.2)	0.009
DLQI difference°[mean(±SD)]	-11.3(15.9)	-4.6(4.6)	0.098
Pain- NRS difference°[mean(±SD)]	-7.7(2.5)	-2.5(3.3)	0.014
Itch- NRS difference°[mean(±SD)]	-3(3.0)	-1.4(1.5)	0.136
HISCR50 n(%)	1(33.3)	7(30.4)	0.919
	Current use of immunomodulant (n=12)	NO current use of immunomodulant (n=45)	P value
IHS4 difference°[mean(±SD)]	-7.0(5.4)	-7.5(5.9)	0.851
DLQI difference°[mean(±SD)]	-5.4(6.4)	-4.9(6.7)	0.873
Pain- NRS difference°[mean(±SD)]	-2.8(2.7)	-2.8(3.7)	0.996
Itch- NRS difference°[mean(±SD)]	-1.8(2.2)	-1.4(1.6)	0.669
HISCR50 n(%)	2(40.0)	7(29.2)	0.634

°Mean difference between baseline and week 12 or week 24 assessed scores
